# Supplementary figures and images for: Protein features for assembly of the RNA editing helicase 2 subcomplex (REH2C) in Trypanosome holo-editosomes
Source: PLoS One. 2019 Apr 29;14(4):e0211525. doi: 10.1371/journal.pone.0211525 (PMC6488192; doi:10.1371/journal.pone.0211525)

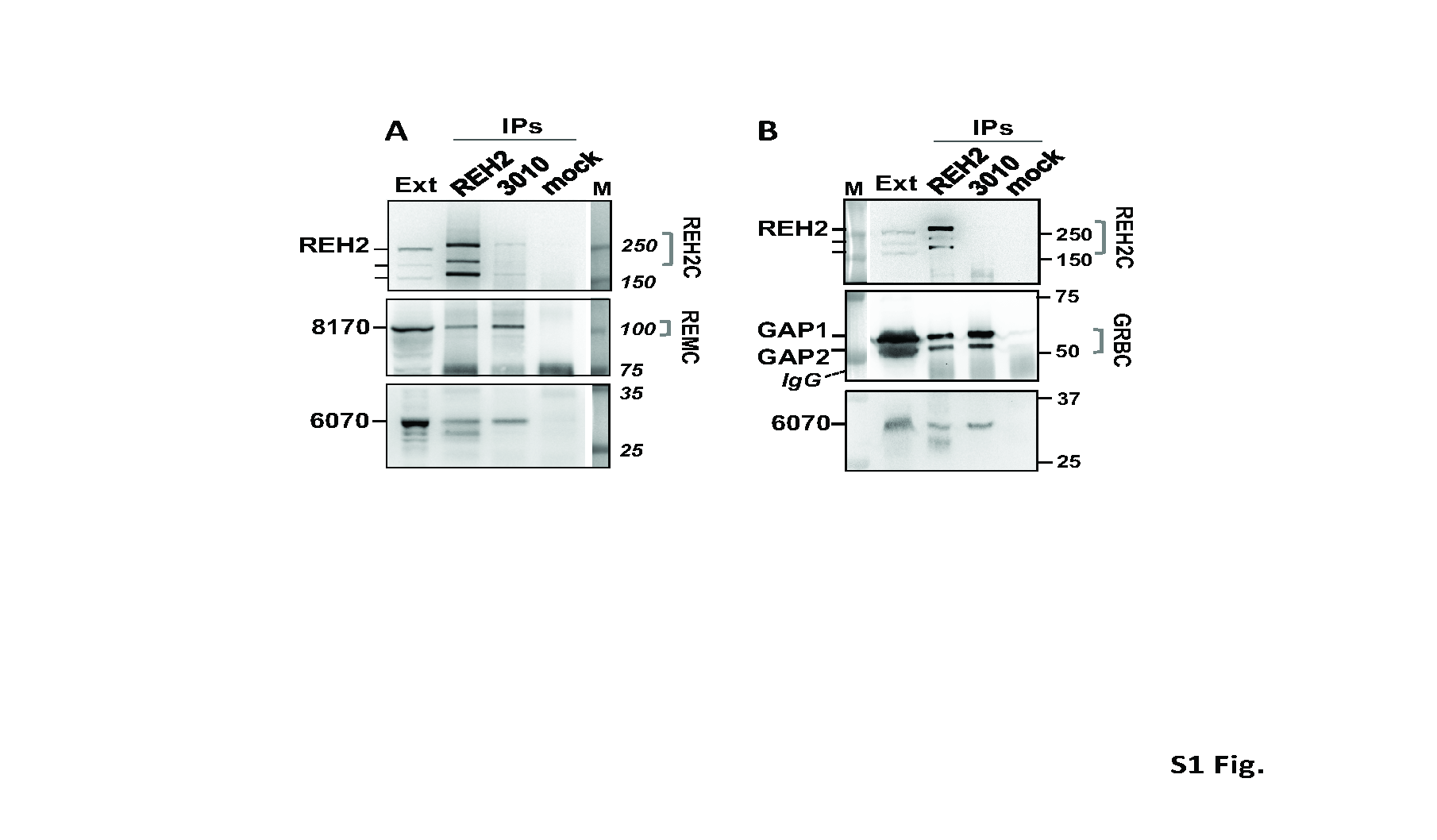

Supplement: S1 Fig — Immunoprecipitations (IP) from extract with specific antibodies against REH2, MRB3010 or CoxI (mock). (A) Western blot analyses examined for the presence of REH2, MRB8170 and MRB6070 proteins. These are markers of REH2C, REMC and a MRB6070/MRB1590-containing subcomplex that associates with RESC, respectively. Extract (Ext) used in the IPs and size markers in kDa (M) are indicated. (B) Western blot analyses as in A examining REH2, the GAP1/GAP2 paralogs, and MRB6070. We note that proteins in the IPs are often upshifted relative to the control extract lane. The cause of this upshift is unclear to us but it may be a slight effect on the proteins migration due to the presence of IgG in those samples. IgG in the pulldown lanes is indicated. (TIFF) [file pone.0211525.s001.tiff]

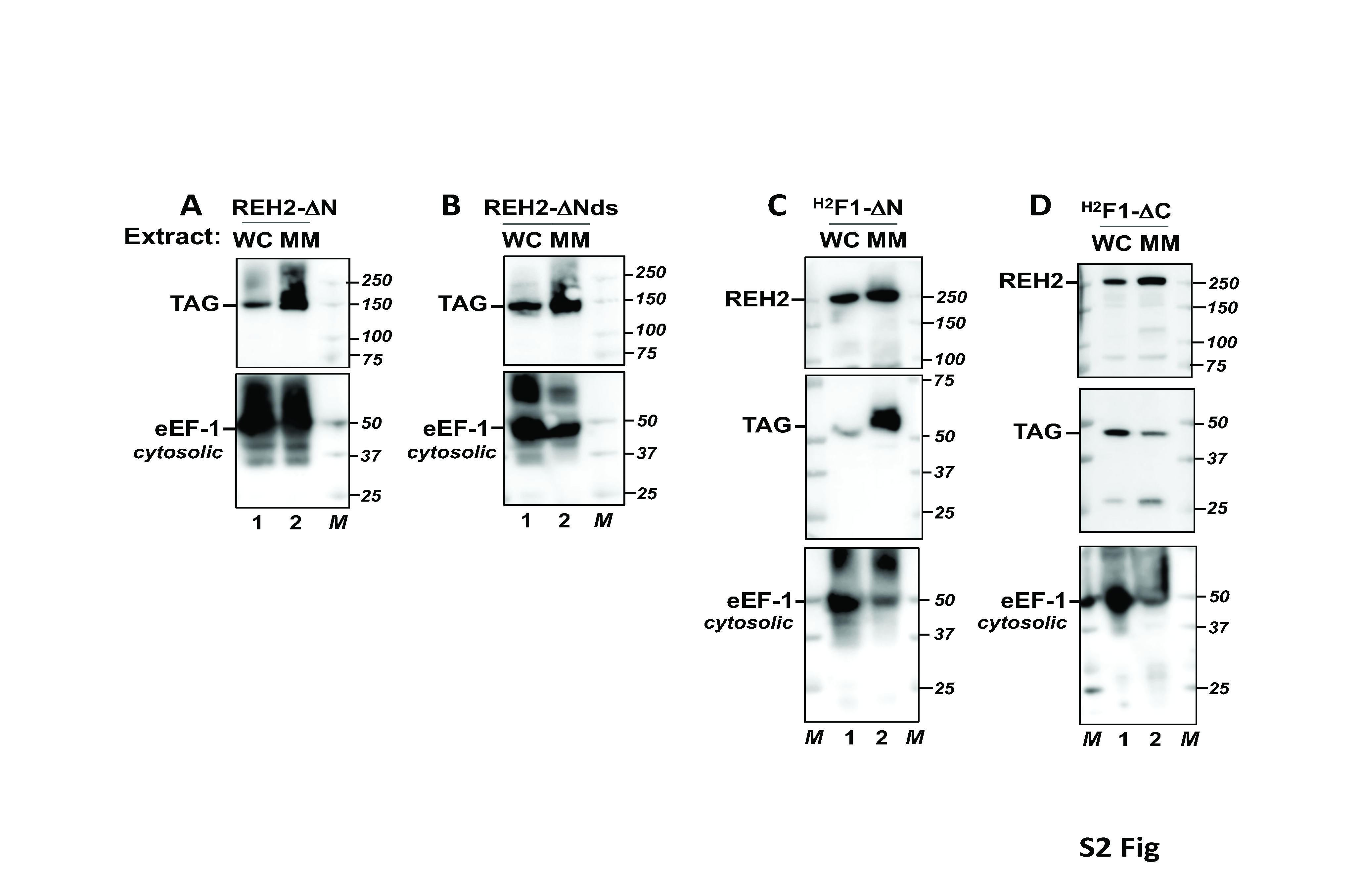

Supplement: S2 Fig — Western blots of large-truncation constructs in whole-cell (WC) and enriched-mitochondrial (aka Mini-Mito “MM” extract): (A) REH2-ΔN, (B) REH2-ΔNds, (C) H2F1-ΔN, and (D) H2F1-ΔC. Western blots of the tag in these constructs (TAP), the cytosolic marker eEF-1 (all panels), and endogenous REH2 (panels C and D) indicate a partial enrichment of the REH2 deletion constructs in the mini-mito extract. Some mitochondrial enrichment of H2F1-ΔC is apparent relative to eEF-1 but the localization of H2F1-ΔC is clearly compromised compared to other constructs examined. (TIFF) [file pone.0211525.s002.tiff]

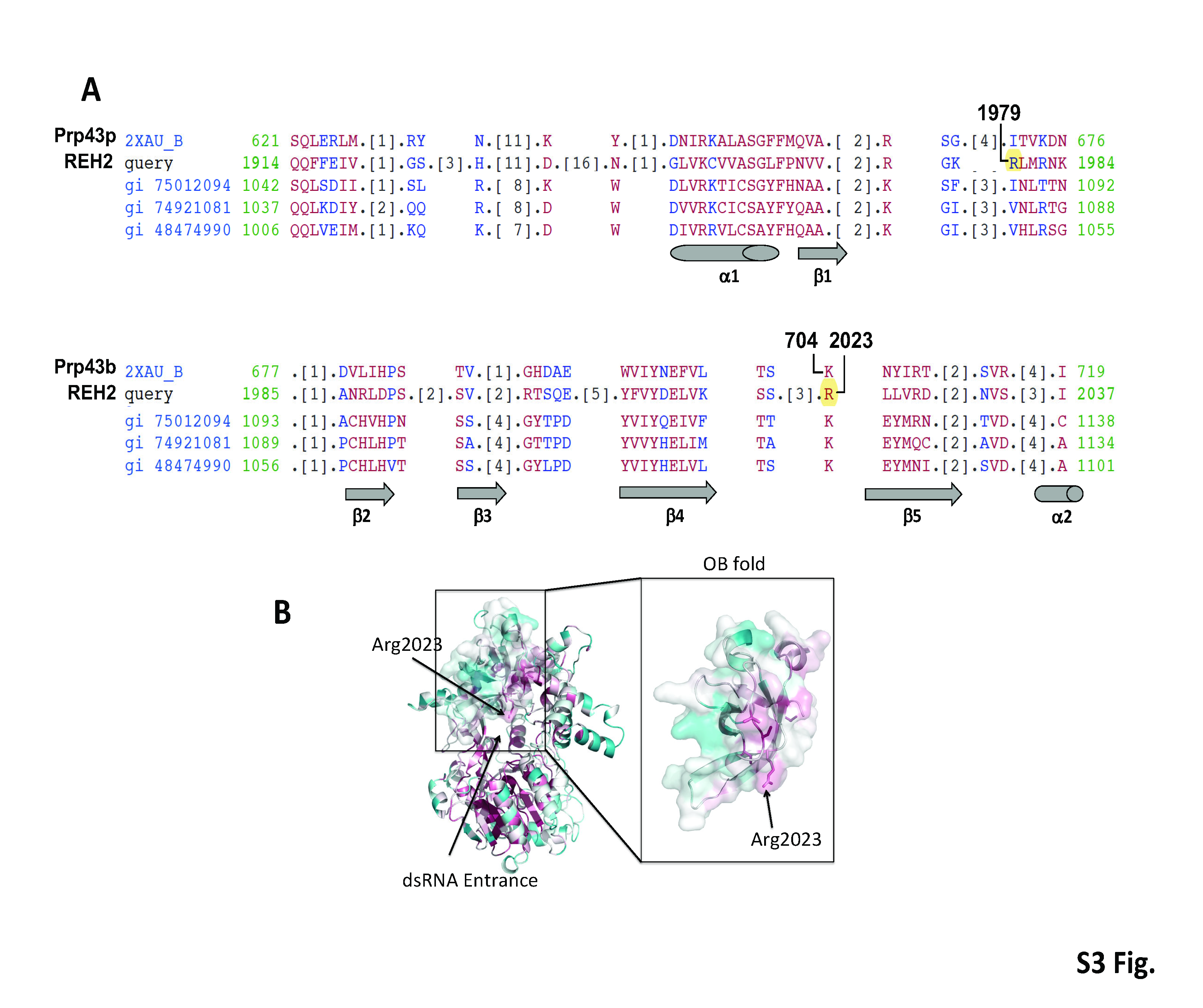

Supplement: S3 Fig — (A) Sequence alignment of Prp43p, REH2, and other DExH/RHA helicases. The sequences were aligned using the CDD tool in NCBI. The residue R1979 in REH2 is not conserved in the aligned sequences but the basic amino acid at position 2023 in REH2 (K704 in Prp43p) is conserved. Secondary structure elements are indicated: α-helix (cylinders) and β-strand (arrows). (B) homology model of R2023 in REH2 using the OB fold in Prp43p as a template. (TIFF) [file pone.0211525.s003.tiff]

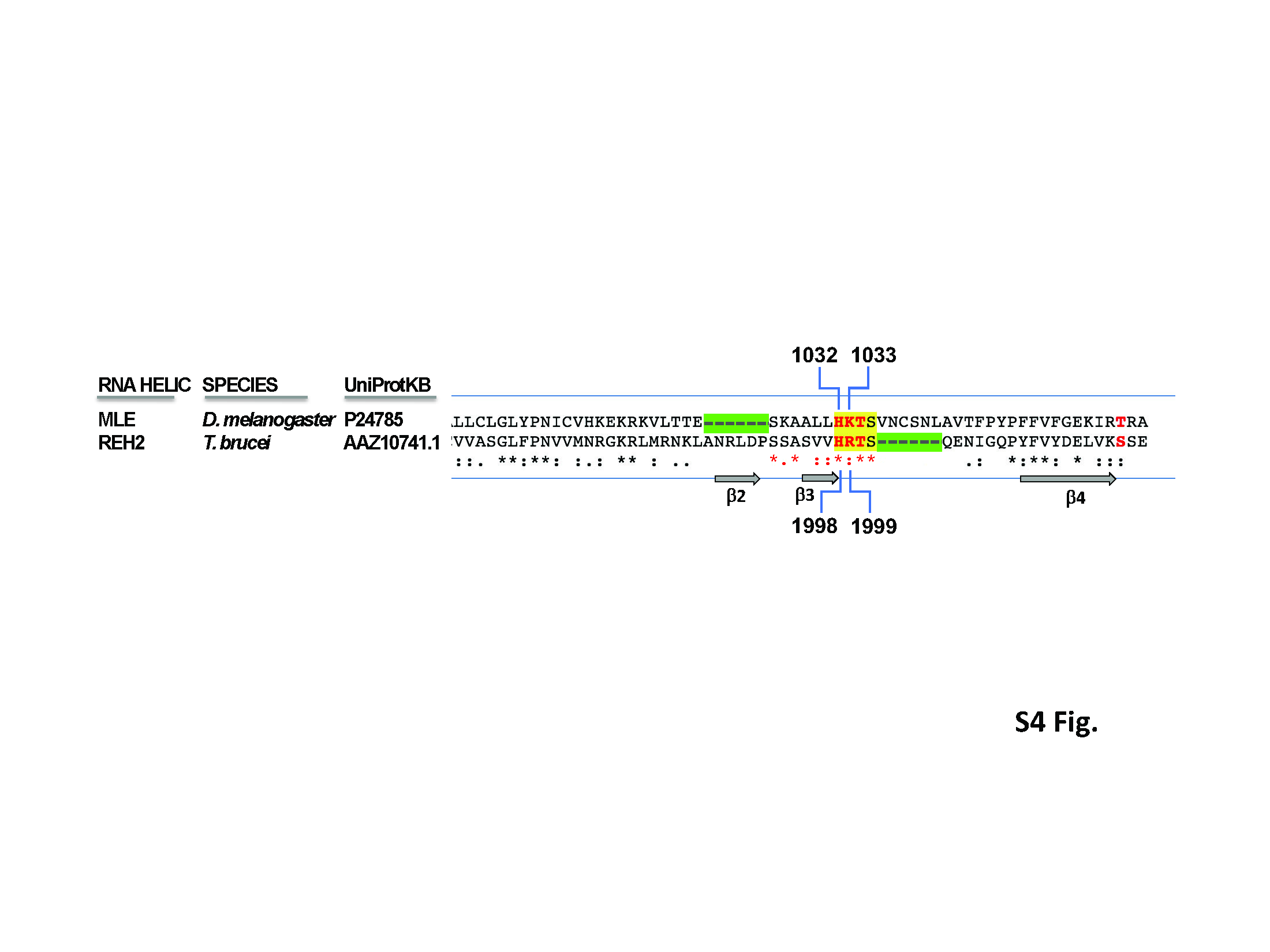

Supplement: S4 Fig — The alignment was generated with Clustal Omega [48]. Boxes were inserted manually to improve the match between the MLE and REH2 residues. Note that H1032, K1033 and T1034 (in red), which make U-specific contacts in MLE, are aligned with H1998, R1999 and T2000 (in red) in REH2. Predicted α-helix (cylinders) and β-strand (arrows) in REH2 are indicated. (TIFF) [file pone.0211525.s004.tiff]

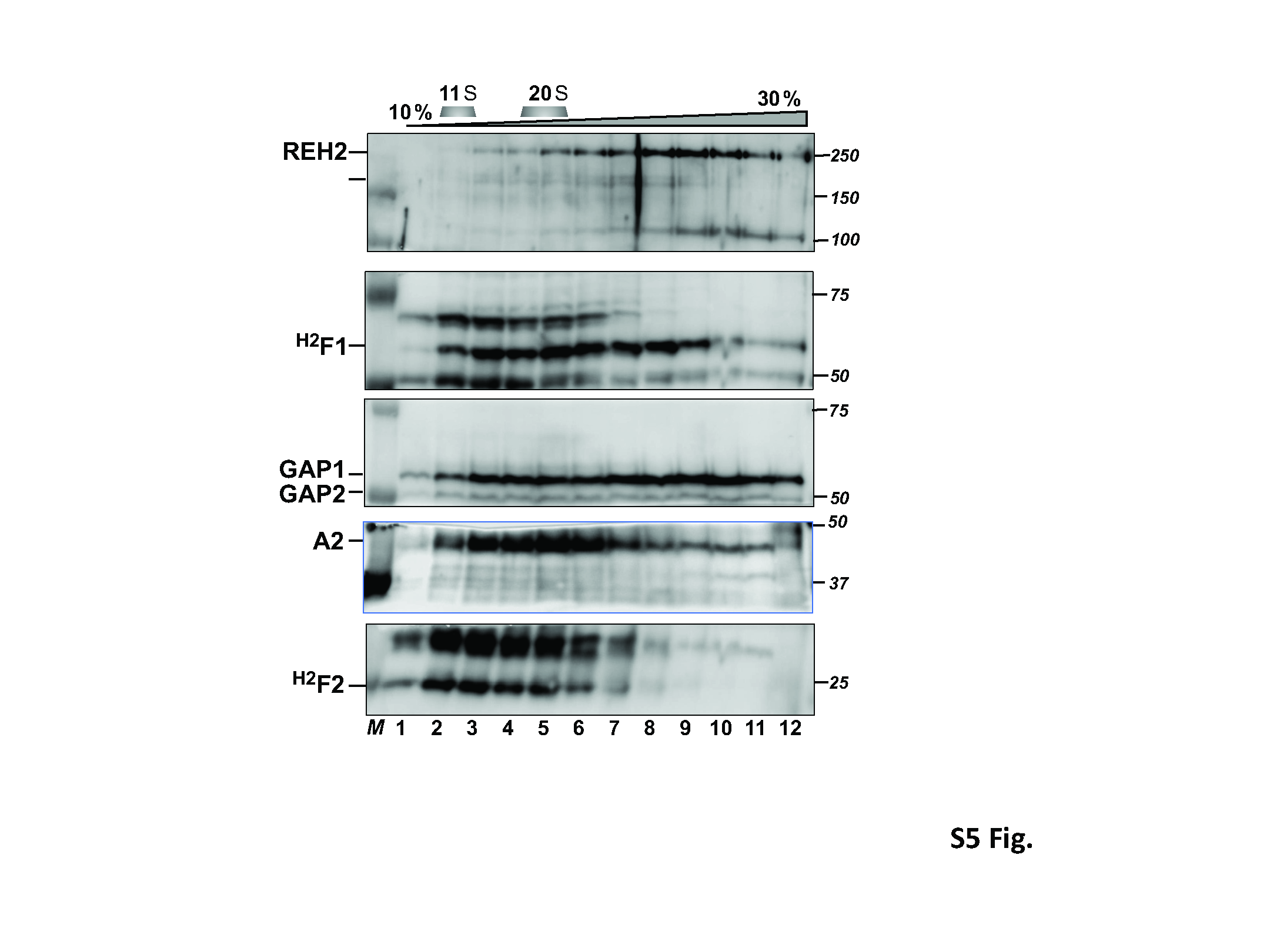

Supplement: S5 Fig — (A) 10–30% glycerol gradients of freshly-made mitochondria-enriched extract from 29:13 procyclic trypanosomes. Catalase and RECC complex were used as 11 S and 20 S markers, respectively [23]. Endogenous REH2, H2F1 and H2F2, GAP1 (GRBC2), GAP2 (GRBC1), and A2 (MP42) were examined in western blots. All panels in this figure derived from the same extract fractions. The data shown is representative of at least two panels for each protein in biological replicate gradients. (TIFF) [file pone.0211525.s005.tiff]

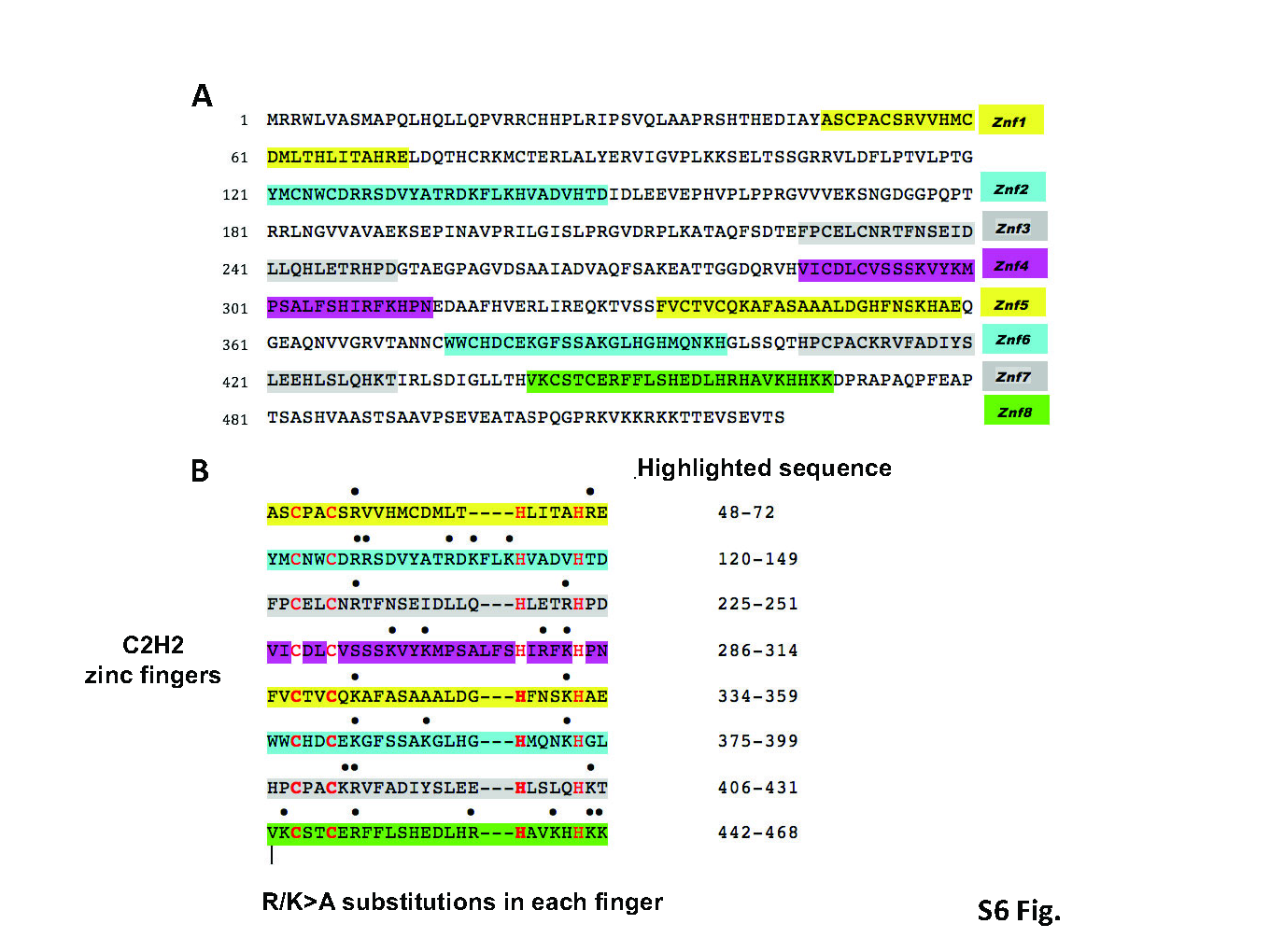

Supplement: S6 Fig — (A) Full H2F1 amino acid sequence including the location of eight C2H2 zinc-finger motifs (Znf1-to-8 highlighted in different colors). (B) Zinc-finger motifs starting with the N terminal finger at the top, and the amino acid positions spanning each finger in panel A. The R/K>A substitutions in each finger that were examined in this study are marked by a dot. (TIFF) [file pone.0211525.s006.tiff]

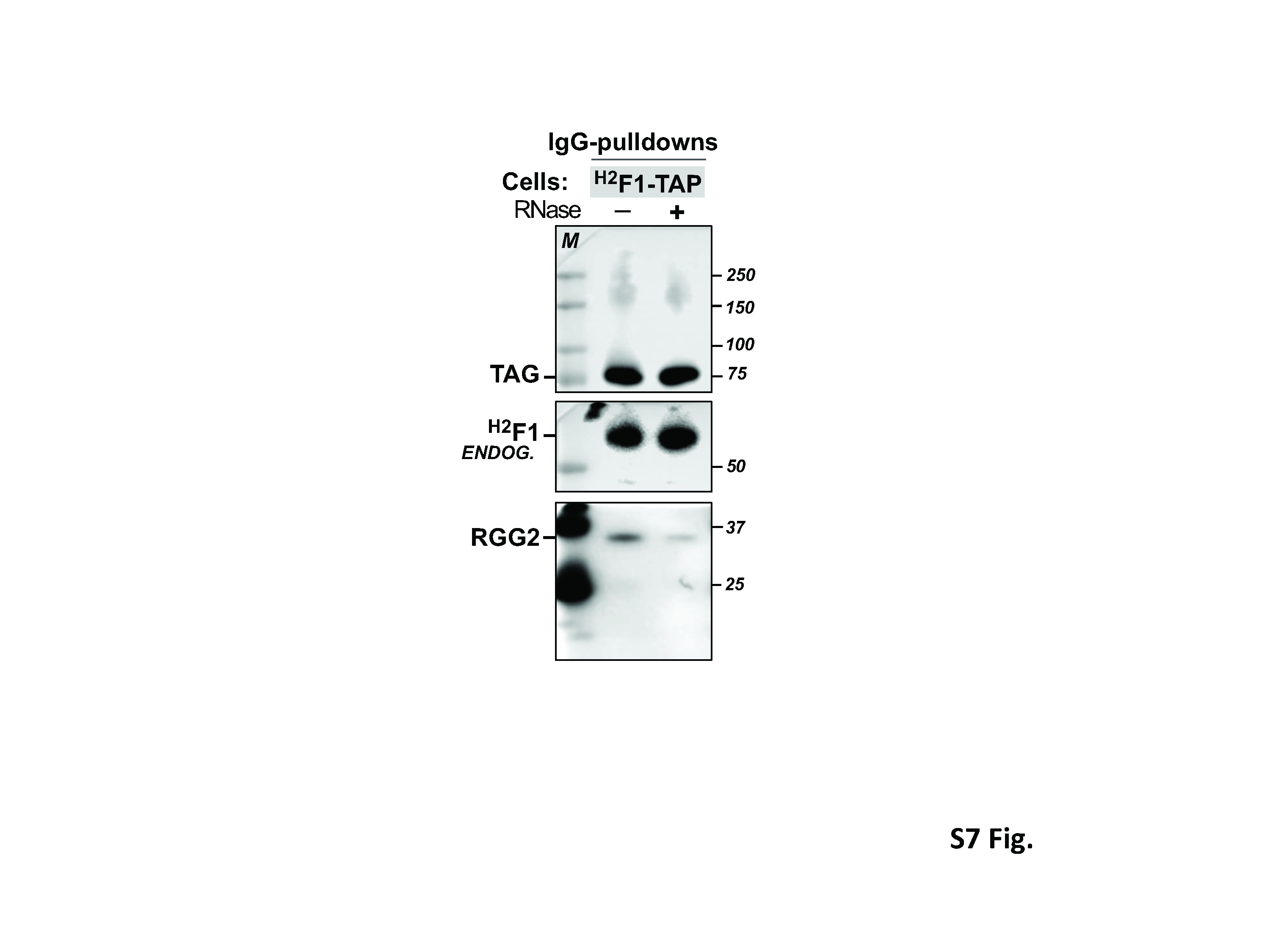

Supplement: S7 Fig — Western blots of IgG pulldowns from extracts with or without an RNaseA/T1 mix. All panels in this figure derive from the same blot. The upper blot with the tagged-H2F1 bait was cut below the 75 kDa marker. The middle and lower panels were divided between the 50 kDa and 37 kDa marker. The 34.4 kDa RGG2, a typical subunit of the REMC module in the RESC complex. As expected, the RNA-mediated association of RGG2 decreased with the RNase treatment. (TIFF) [file pone.0211525.s007.tiff]

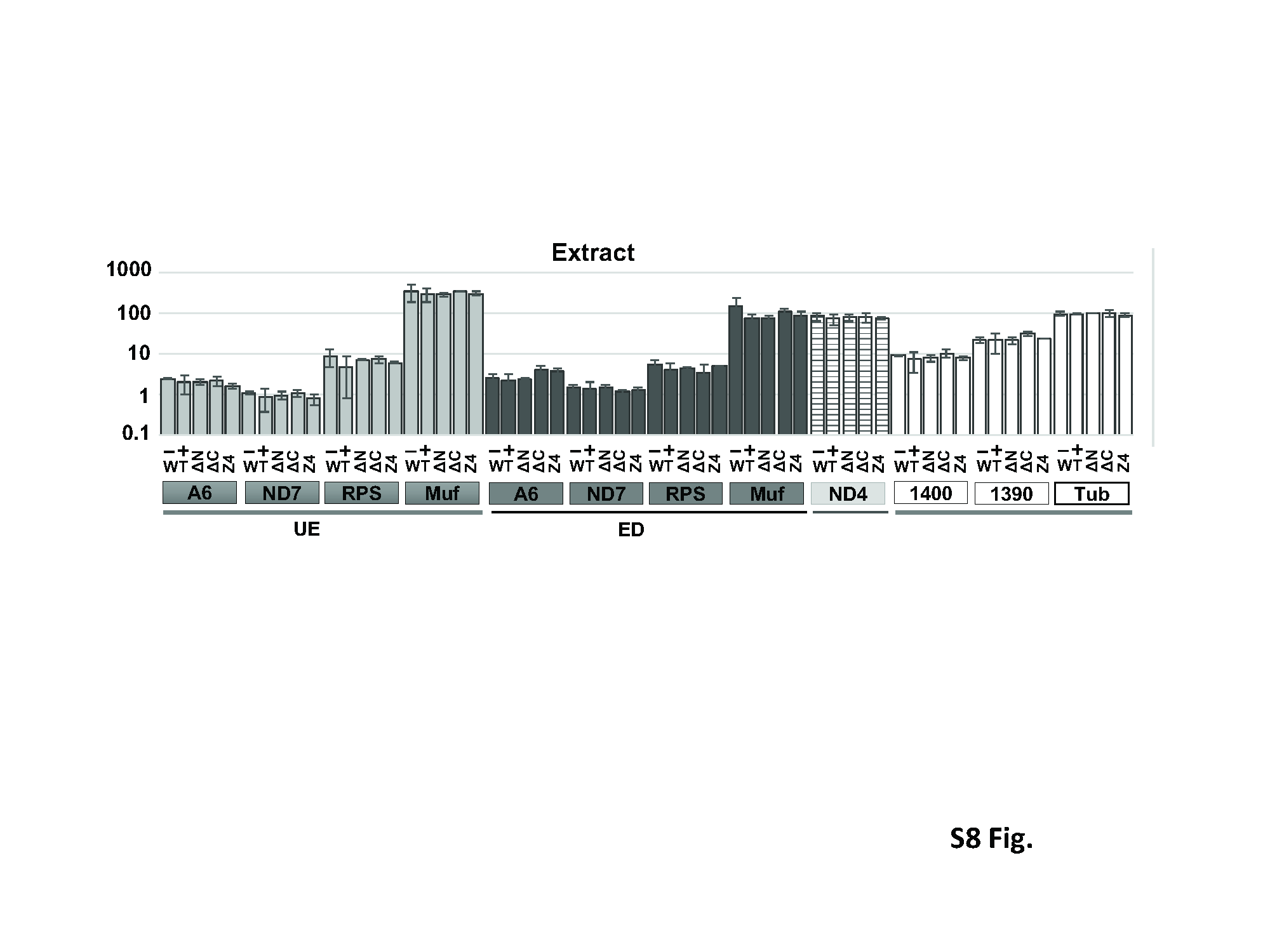

Supplement: S8 Fig — Independent biological replicates (two independent cultures used in Fig 5) with Cq average values and one standard deviation (+/-1SD, n = 2) were plotted. dCq of steady-state RNA transcripts in lysates relative to background 18s rRNA used as reference [dCq = 2(target Cq–ref Cq)]. Shorter bars indicate a smaller differential versus 18s rRNA in the sample. For example, ND7 is relatively abundant compared to other transcripts in the sample (i.e., it has a lower Cq). The WT construct is induced or not (+/-). All mutants are induced. All end-point amplicons were examined in gels to confirm that they were single products during linear amplification. Abbreviation of the RNA names is as in Fig 5B. (TIFF) [file pone.0211525.s008.tiff]
