# Supplementary material for: Protein features for assembly of the RNA editing helicase 2 subcomplex (REH2C) in Trypanosome holo-editosomes
Source: PLoS One. 2019 Apr 29;14(4):e0211525. doi: 10.1371/journal.pone.0211525 (PMC6488192; doi:10.1371/journal.pone.0211525)
Supplement: S1 Table — (PDF) [file pone.0211525.s009.pdf]

**REH2 mutant constructs**				**Reference**
dsR (dsRBD2 K1078A/K1086A)				
Two-step site-directed mutagenesis	F-1045	GGCGTAGCGTGGAAT**GCA**GAGGCCTCGCAACGC		3
	R-1046	GCGTTGCGAGGCCTC**TGC**ATTCCACGCTACGCC		
K1078A primers, PCR #1	F-1047	GCAACGCCAGGCG**GAC**ATGCACGCC		
K1086A primers, PCR #2	R-1048	GGCGTGCAT**GTC**CGCCTGGCGTTGC		
ΔN (keeps dsRBD2)				
Two fragment In-Fusion	F-1503	CACAAGCTTCTCGAGATGCGGGCCATACGACTAAC		
	R-1522	CTGCGTTTGAAACAGTCGCACC		This study
	F-1523	CTGTTTCAAACGCAGGATGCAAAAACAGTGTTGCAGCGTTAC		
Deletion: 989 aa (Interval: 103-3069 nt)	R-1511	CTTTTCCATGGATCCCGAGTCTCCACCAGCCTC		
ΔNds (removes dsRBD2)	F-1503	CACAAGCTTCTCGAGATGCGGGCCATACGACTAAC		
Two fragment In-Fusion	R-1522	CTGCGTTTGAAACAGTCGCACC		This study
	F-1524	CTGTTTCAAACGCAGCGCCGTGTAGAACCAGATTTCGC		
Deletion: 1059 aa (Interval 103-3279 nt)	R-1511	CTTTTCCATGGATCCCGAGTCTCCACCAGCCTC		
ΔAOB (keeps OB)	F-1503	CACAAGCTTCTCGAGATGCGGGCCATACGACTAAC		This study
Deletion: 123 aa (Interval: 6133-6501 nt)	R-1504	CTTTTCCATGGATCCGCTGCTGGTGCCCATTAA		
ΔOB (removes OB)	F-1503	CACAAGCTTCTCGAGATGCGGGCCATACGACTAAC		This study
Deletion: 260 aa (Interval: 5723-6501 nt)	R-1505	CTTTTCCATGGATCCAAGGAGCTGAGGGACGC		
R1979A (OB)	F-1464	GTTATGAACCGGGGGAAG**GCC**CTCATGAGG		This study
	R-1465	CCTCATGAG**GGC**CTTCCCCCGGTTCATAACGACATTAGG		
H1998E (OB)	F-1571	TCTGTTGTC**GAG**CGTACATCACAGGAAAATAATG		This study
	R-1572	TGTACG**CTC**GACAACAGATGCCGATGATGGGTCCAG		
R1999E (OB)	F-1573	TCTGTTGTCCAT**GAG**ACATCACAGGAAAATATTG		This study
	R-1574	TGATGT**CTC**ATGGACAACAGATGCCGATGATGGGTCCAG		
H1998E/R1999E (OB)	F-1602	TCTGTTGTC**GAGGAG**ACATCACAGGAAAATATTG		This study
	R-1603	CTGTGATGT**CTCCTC**GACAACAGATGCCGATG		
R2023A (OB)	F-1470	GGAATCCGAA**GCG**CTGCTCGTG		This study
	R-1469	CACGAGCAG**CGC**TTCGGATTCC		
**H2F1 WT and mutant constructs**				
H2F1 WT	F-1487	CATACATAAAGCTTATGCGGCGCTGGTTGGTGGC		This study
	R-1490	ATCAGCAGGATCCCGACGTCACCTCACTTACC		
Canonical cysteine residues				
Z5 C-to-A	F-1582	GTTTGCCAGGCGGCTTTCGCTTCCGCTG		This study
	R-1583	AGCGAAAGCCGCCTGGCAAACCGTGC		
Primers PCR#1	F-1584	CTTTAATAGCGCGCATGCGGAACAAGG		
Primers PCR#2	R-1585	GTTCCGCATGCGTATTAAAGTGTCC		
Variable basic residues				
Z1 R/K-to-A	1590	gBlock	Sequences available	This study
Z2 R/K-to-A	1591	gBlock	upon request	
Z3 R/K-to-A	1592	gBlock		
Z4 R/K-to-A	1622	gBlock		
Z5 R/K-to-A	1623	gBlock		
N- and C- truncations				
ΔN	F-1503	CACAAGCTTCTCGAGATGCGGGCCATACGACTAAC		This study
Two fragment In-Fusion	R-1522	CTGCGTTTGAAACAGTCGCACC		
The MLS derives from REH2 (see footnote)	F-1609	CTGTTTCAAACGCAGGGGCCCGCAGGTGTTGAC		
Deletion: 255 aa (Interval: 1-765 nt)	R-1608	CTTTTCCATGGATCCGGGCCCCTCTGCAGT		
ΔC	F-1607	CACAAGCTTCTCGAGATGCGGCGCTGG		This study
Deletion: 267 aa (Interval: 772-1572 nt)	R-1608	CTTTTCCATGGATCCGGGCCCCTCTGCAGT		
**mRNA/gRNA duplex**				
gRNA				
Template in PCR	1354	AAGCAGAAGAGATACGTTTAAAAAAAAAAAAAAAATTATCATACCACTGTAAA		
		ACTGATTTCGTCATTGGAGTTATAGTTATATCCTATAGTGAGTCGTA		23
Primers in PCR	F-1356	AAGCAGAAGAGATACGTT		
	R-1385	TAATACGACTCACTATAGGATATACTATAAC		
mRNA	566	GAGAGAGGAGAGAAGAAAGGGAAAGUUGUGAUUUUGGAGUUAUAGAAUACUUACCUGGCAUC		23
Synthetic fragment mA6 11-72				
**Cloning recombinant REH2**				
	F-REH2 &	AGAAGGAGATATACCATGGCCATGTTTCAAACGCAGGAAATTAC		This study
Amino acids 30-2167	R-REH2 #	GGCTTTGTTAGCAGCCGGATCCTCATTAGTGATGGTGATGGTGATGCGGCGAG		
		TCTCCACCAGCCTCAGC		
& full name: pET15bNCO1REH2fwd				
# full name: pET15b2stop6HisProlineREH2Rev				
MLS: mitochondrial leader sequence fragment (Primers: F-1503, R-1522)				
The undeline indicates the mutated nucleotides in the indicated primers.				
